# Supplementary material for: Unraveling the hidden temporal range of fast β2-adrenergic receptor mobility by time-resolved fluorescence
Source: Commun Biol. 2022 Feb 28;5:176. doi: 10.1038/s42003-022-03106-4 (PMC8885909; doi:10.1038/s42003-022-03106-4)
Supplement: Supplementary file 2 — Supplementary Information [file 42003_2022_3106_MOESM2_ESM.pdf]

Supplementary information for

Unraveling the hidden temporal range of fast β_2-adrenergic receptor mobility by time-resolved fluorescence

Ashwin Balakrishnan[1#], Katherina Hemmen[1#], Susobhan Choudhury[1], Jan-Hagen Krohn[1], Kerstin Jansen[1], Mike Friedrich[1], Gerti Beliu[1], Markus Sauer[1,2], Martin J. Lohse[3]*, Katrin G. Heinze[1]*

*Martin J. Lohse, Katrin G. Heinze
Email: lohse@toxi.uni-wuerzburg.de, katrin.heinze@virchow.uni-wuerzburg.de

This PDF file includes:

Figures S1 to S16
Tables S1 to S6
SI References

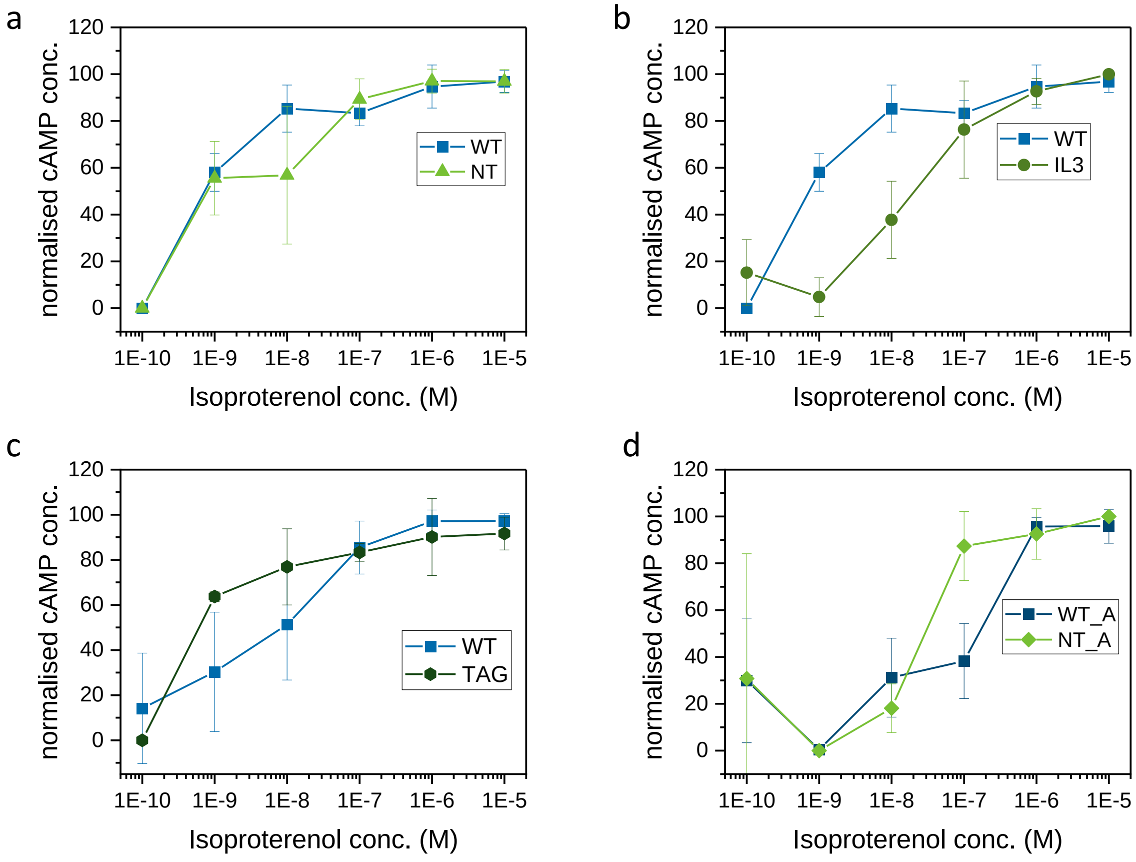

Supplementary Figure 1. cAMP response upon ligand activation. cAMP response was measured using the commercial Abcam fluorimetric cAMP assay (see Methods) with activation using increasing concentrations of ligand (Isoproterenol) ranging from 100 pM to 10 µM. . **(a)** WT and NT **(b)** WT and IL3 **(c)** WT and TAG **(d)** WT_A and NT_A. WT data shown in (a) and (b) are the same. All cases except (c) were expressed in CHO-K1 cells. Both WT and TAG in (c) were expressed in HEK 293T cells. Datapoints for each concentration were acquired from three independent experiments. EC_{50} values of (a-d) are given in Supplementary Table 1**.**

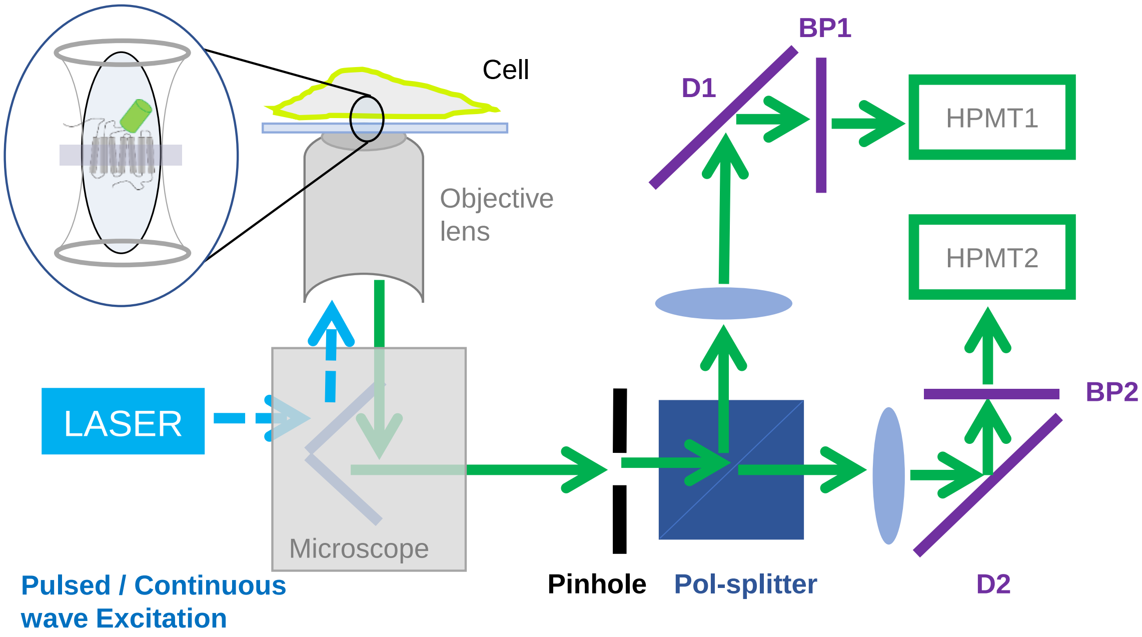

Supplementary Figure 2. Homebuilt confocal TCSPC setup. Schematic of the two channel Fluorescence Correlation Spectroscopy setup. The pulsed laser light is fed into the microscope and targeted through the objective onto the basal membrane of the cell. The emitted fluorescence is collected through the same objective, guided through a pinhole to suppress out-of-focus fluorescence and split into its vertical and horizontal components by a polarizing beam splitter (Pol-splitter). The fluorescence is further cleaned by dichroic beamsplitters (D1, D2) and bandpass filters (BP1, BP2) for the green fluorescence. More technical details are described in the Methods section.

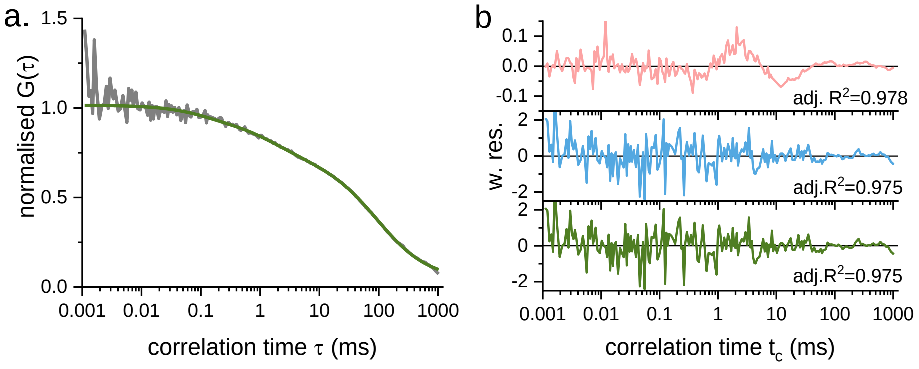

Supplementary Figure 3. Fitting reveals multiple diffusion times. (a) Representative autocorrelation curve of NT fitted with Model 2D3DT (**eq. 4**) showing a photophysics-related component (or relaxation time) of 13.03 ± 3.03 µs, and two diffusion times of 1.15 ± 0.07 ms and 88.76 ± 4.32 ms. **(b)** Weighted residuals of different models used to fit the ACF in (a): 2D2T (red), 22DT (blue) and 2D3DT (green). The corresponding adj. R^2 are also stated.

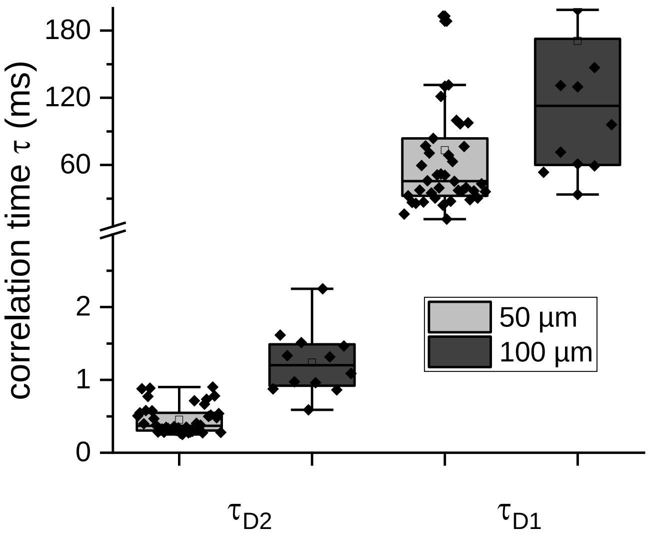

Supplementary Figure 4. Fast diffusion time is not an artefact from photophysics. Diffusion times obtained from fitting data measured using different pinhole sizes effectively changing the focal volume. 50 µm pinhole measurement shows τ_{d1} = 0.45 ± 0.19 ms and τ_{d2} = 73.04 ± 71.41 ms while 100 µm pinhole measurement shows τ_{d1} = 1.23 ± 0.44 ms and τ_{d2} = 170.73 ± 189.59 ms.

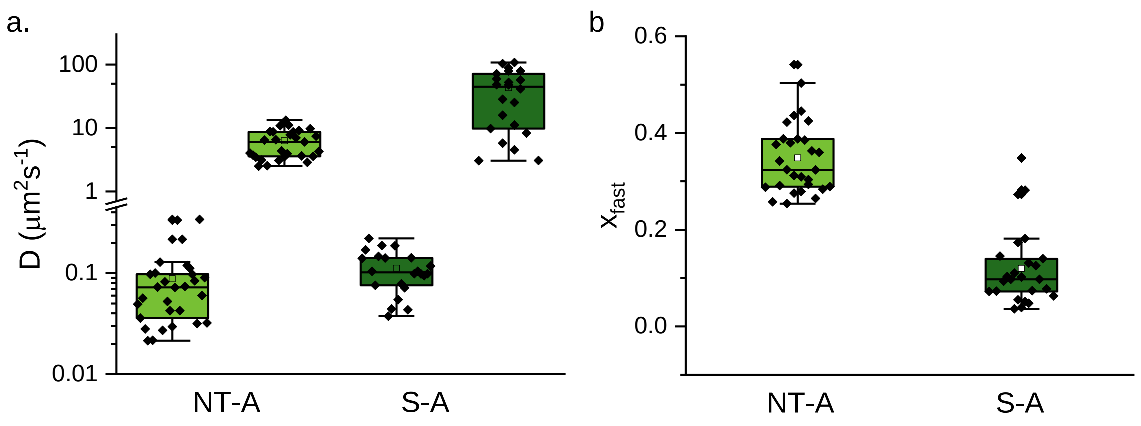

Supplementary Figure 5. α_{2A}-AR shows similar translational diffusion as β_2-AR. (a) Diffusion constants calculated from fluorescence intensity measurements of CHO-K1 cells expressing NT-A and S-A. NT-A shows D_{fast} = 6.41 ± 2.97 μm^{2}s^{-1} and D_{slow} = 0.08 ± 0.06 μm^{2}s^{-1}; S-A shows D_{fast} = 40.06 ± 33.56 μm^{2}s^{-1} and D_{slow} = 0.10 ± 0.05 μm^{2}s^{-1}. (b) Fraction of the fast diffusion component corresponding to the data in (a). NT-A shows 0.34 ± 0.07 while S-A shows 0.10 ± 0.05.

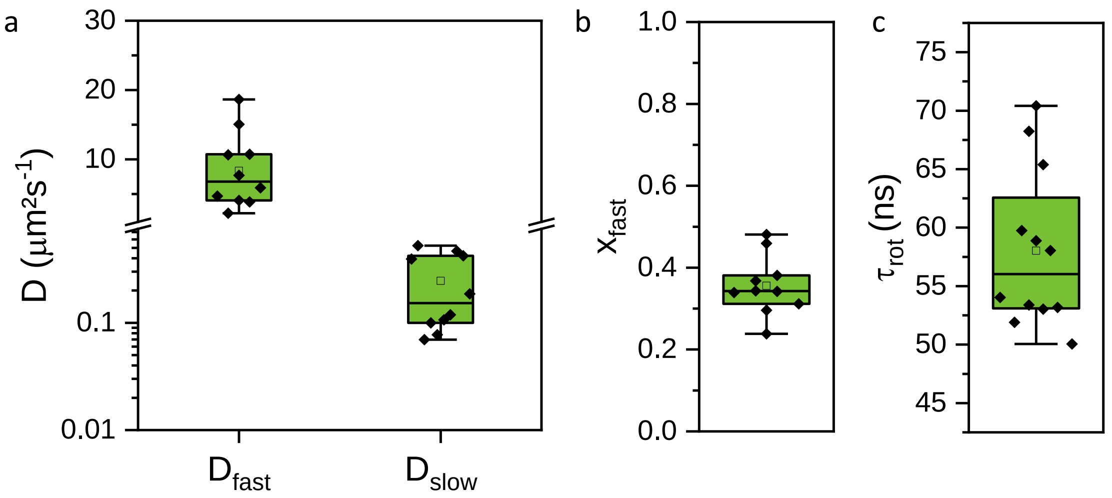

Supplementary Figure 6. NT-β$_2$-AR expressed in HEK 293T cells exhibits similar diffusion parameters as expressed in CHO-K1 cells. (a) Diffusion constants of NT expressed in HEK 293T cells, D_{fast} = 8.35 ± 5.34 μm^{2}s^{-1} and D_{slow} = 0.24 ± 0.18 μm^{2}s^{-1}. (b) Fraction of fast diffusion constant corresponding to the data in (a), x_{fast} = 0.36 ± 0.07. (c) Rotational correlation time of NT expressed in HEK 293T cells, τ_{rot} = 58 ± 7 ns.

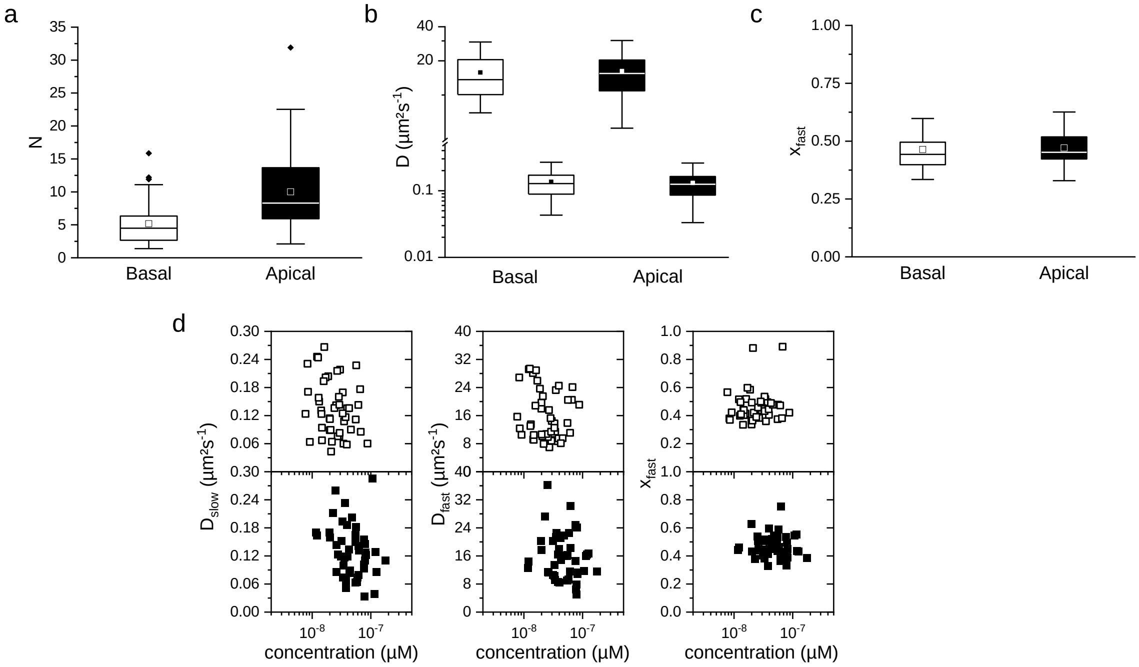

Supplementary Figure 7. Diffusion times are independent of focus position. (a) Number of molecules in the basal (5.2 ± 3.2) and apical membrane (10.0 ± 6.0). **(b)** Diffusion constants of CHO-K1 cells expressing NT with the focal spot positioned on the basal plasma membrane and the apical plasma membrane. Basal membrane shows D_{fast} = 16.28 ± 6.95 µm^{2}s^{-1} and D_{slow} = 0.17 ± 0.24 µm^{2}s^{-1}. Apical membrane shows D_{fast} = 16.84 ± 8.14 µm^{2}s^{-1} and D_{slow} = 0.34 ± 1.13 µm^{2}s^{-1}. **(c)** Fraction of the molecules exhibiting fast diffusion for data acquired from basal and apical plasma membrane. Basal membrane measurement shows x_{fast} = 0.48 ± 0.14 and apical membrane measurement shows x_{fast} = 0.48 ± 0.10. **(d)** The mobility measures D_{fast}, D_{slow} and x_{fast} show no dependence on the receptor density in the measured concentration range of 10 nM – 200 nM for the apical and basal membrane comparison.

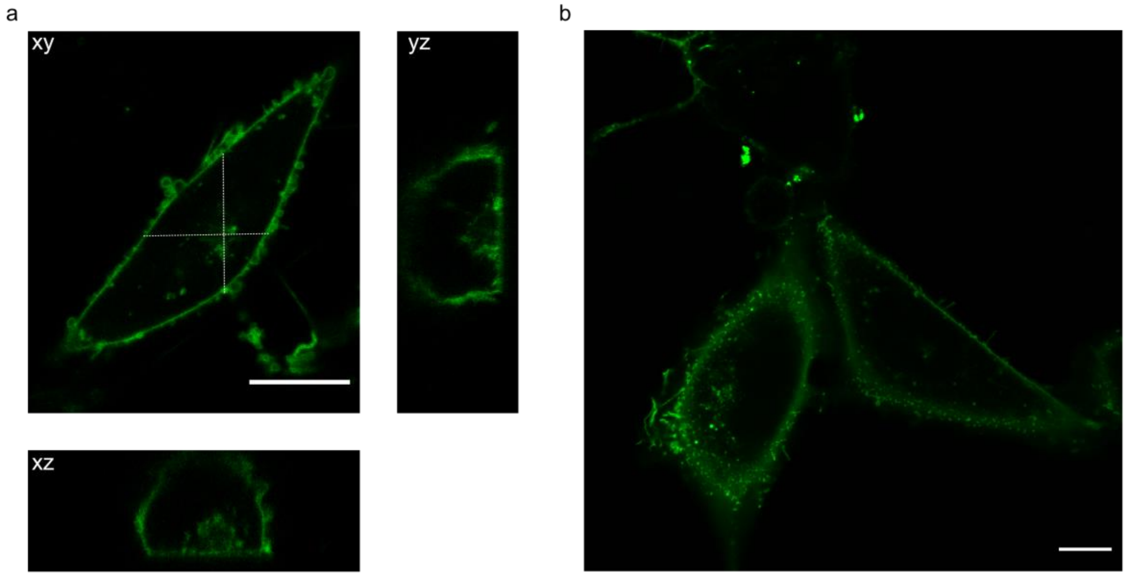

Supplementary Figure 8. AF488 fluorescence is bound to cells. CHO-K1 cells transfected with S receptor are visualised using laser scanning confocal microscopy post labelling with the SNAP tag substrate bound to AF488. (a) Orthogonal projection of a z-stack showing the fluorescent S receptors on the membrane and faintly inside the cell. The vertical dotted white line represents the location of the yz projection and the horizontal line represents the location of the xz projection. (b) A slice of CHO-K1 cells expressing S receptors, showing both membrane and cytoplasm and the localization of fluorescence. Scale bar is 10 µm.

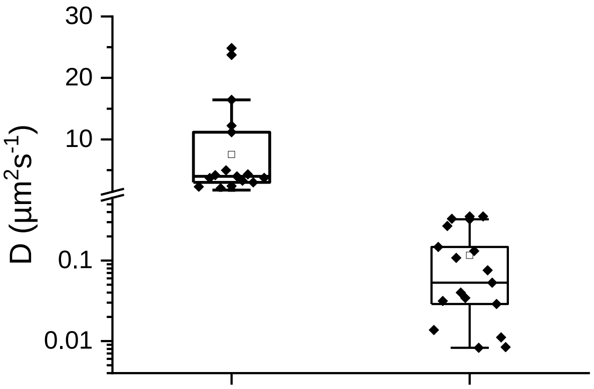

Supplementary Figure 9. Diffusion constants do not change with axial focus position. Diffusion constants of CHO-K1 cells expressing NT with the focal spot axially displaced from basal to the apical plasma membrane. Data from different focal positions are plotted together. The cells exhibit D_{fast} = 7.54 ± 7.51 $\mu m^2 s^{-1}$ and D_{slow} = 0.11 ± 0.12 $\mu m^2 s^{-1}$.

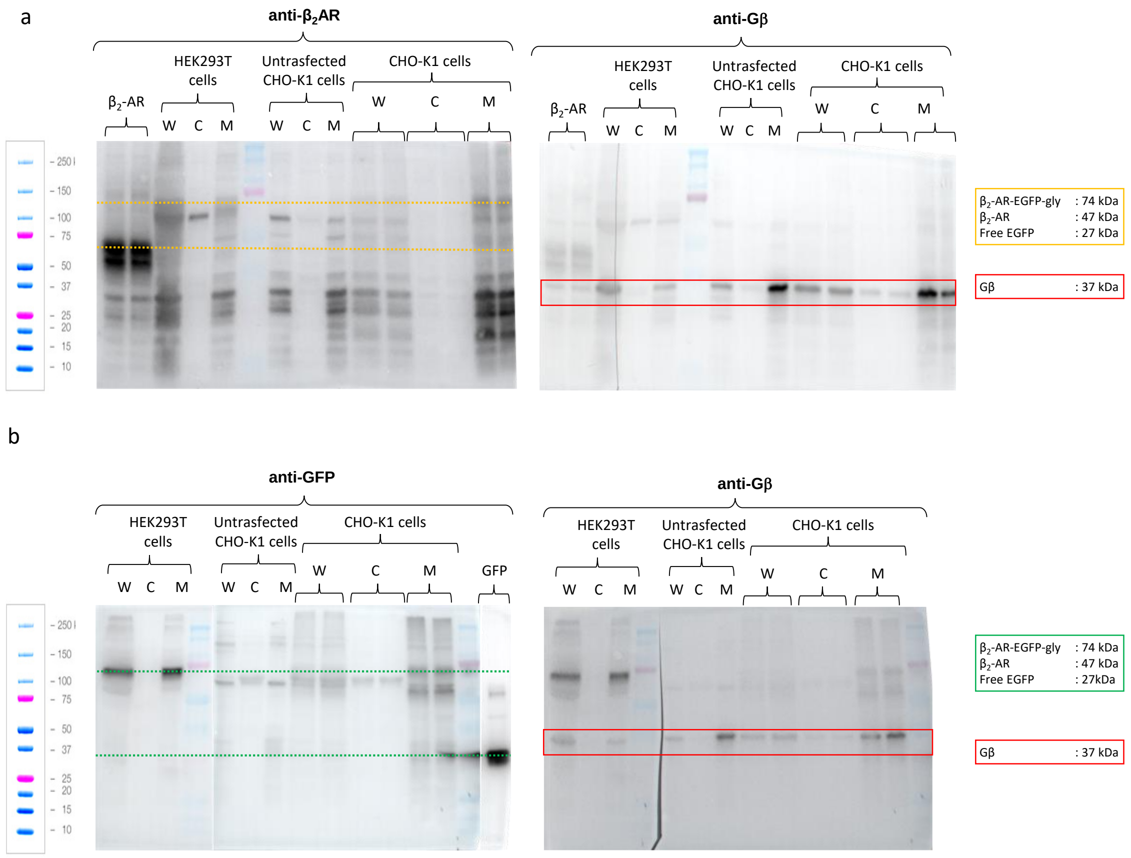

Supplementary Figure 10. Western blot of NT, EGFP-β2-AR. HEK293T cells and CHO-K1 cells were transiently transfected with NT and the whole lysate (W), the cytosolic (C) and the membrane fraction (M) were blotted against anti-β2-AR (a, left) and anti-GFP antibody (b, left). Right panels show the same blot against Gβ, which served as loading control. Please note that the GFP lane is not shown in the anti-Gβ staining (b, right) as the purified protein would not show a band for Gβ. The positive control for the anti-β2-AR antibody was the membrane fraction of HEK293T cells transiently transfected with wild type β2-AR [1]. As a positive control for the anti-GFP antibody we used purified GFP. Expected band sizes are listed on the right and relevant sizes are marked on the blots for easier orientation.

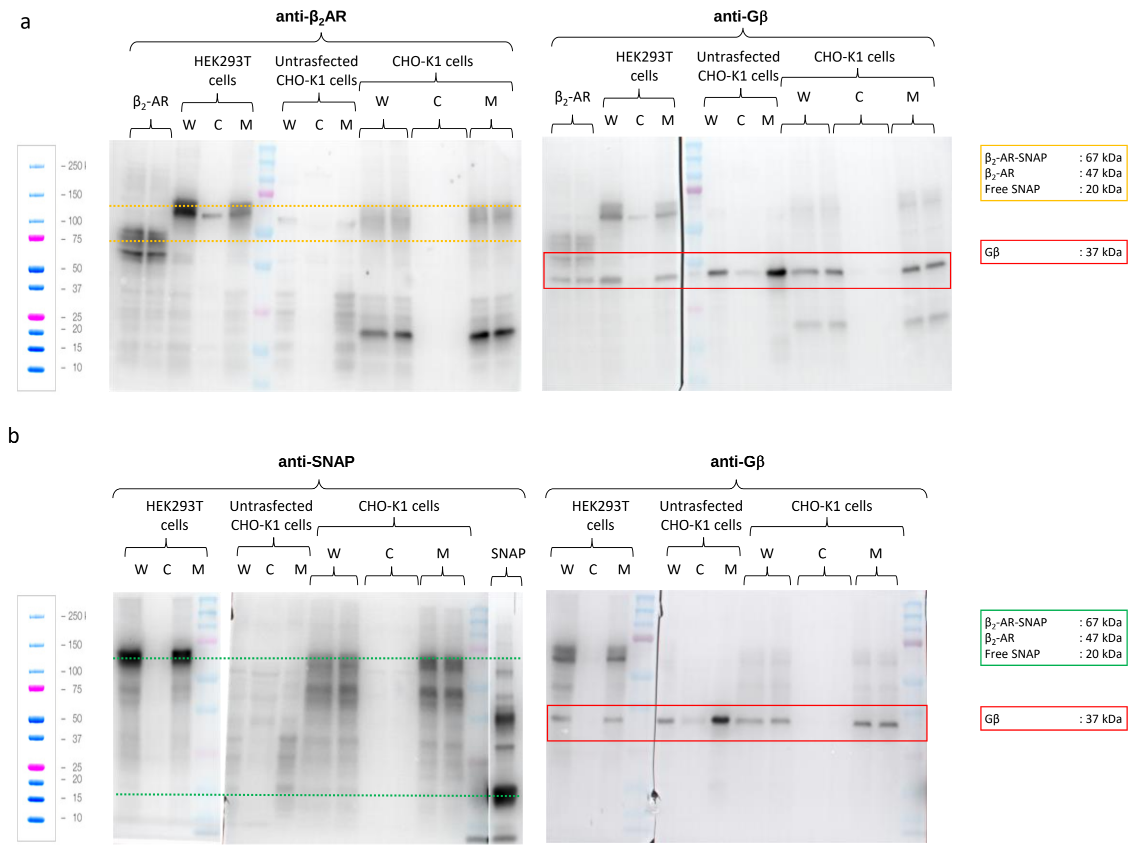

Supplementary Figure 11. Western blot of S, SNAP- β$_2$-AR. HEK293T cells and CHO-K1 cells were transiently transfected with NT and the whole lysate, the cytosolic and the membrane fraction were blotted against anti-β$_2$-AR (a, left) and anti-SNAP-tag antibody (b, left). Right panels show the same blot against Gβ, which served as loading control. The positive control for the anti-β$_2$-AR antibody was the membrane fraction of HEK293T cells transiently transfected with wild type β$_2$-AR [1]. As a positive control for the anti-SNAP tag antibody we used purified GFP. Expected band sizes are listed on the right and relevant sizes are marked on the blots for easier orientation.

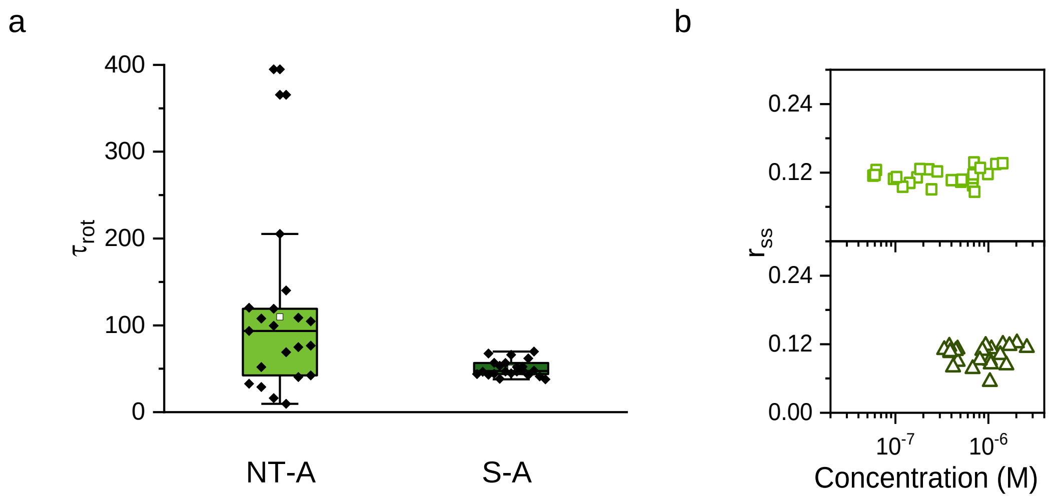

Supplementary Figure 12. TRA reveals dynamics of α_{2A}-AR in line with that of β_2-AR. (a) Rotational correlation times from fitting photon arrival histograms of NT-A and S-A. NT-A shows 81 ± 48 ns and S-A shows 37 ± 15 ns. (b) Steady state anisotropy, r_{ss}, of NT-A and S-A is independent of receptor density. Empty squares represent NT-A and empty triangles represent S-A.

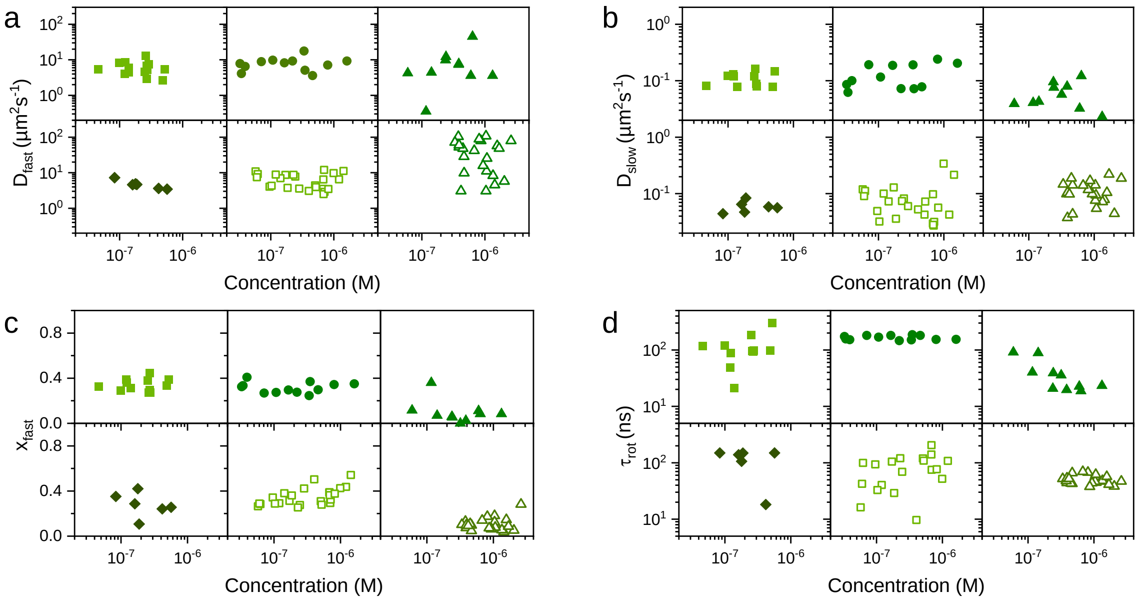

Supplementary Figure 13. Concentration does not seem to influence receptor mobility in both the cases of β2-AR and α2A-AR. The mobility measures (fast and slow diffusion coefficient (a, b), fraction of fast diffusion (c) and rotational correlation time (d)) of all β2-AR and α2A-AR constructs show largely no dependence on the receptor density in the measured concentration range of 20 nM – 5 µM. The only exception might be the fast diffusion coefficient of the β2-AR TAG construct ((a), lower left, dark green diamonds) and rotational correlation time of the β2-AR S construct ((d), upper right, green triangles). Squares represent NT, circles represent IL3, triangles represent S, diamonds represent TAG, Empty squares represent NT-A and empty triangles represent S-A.

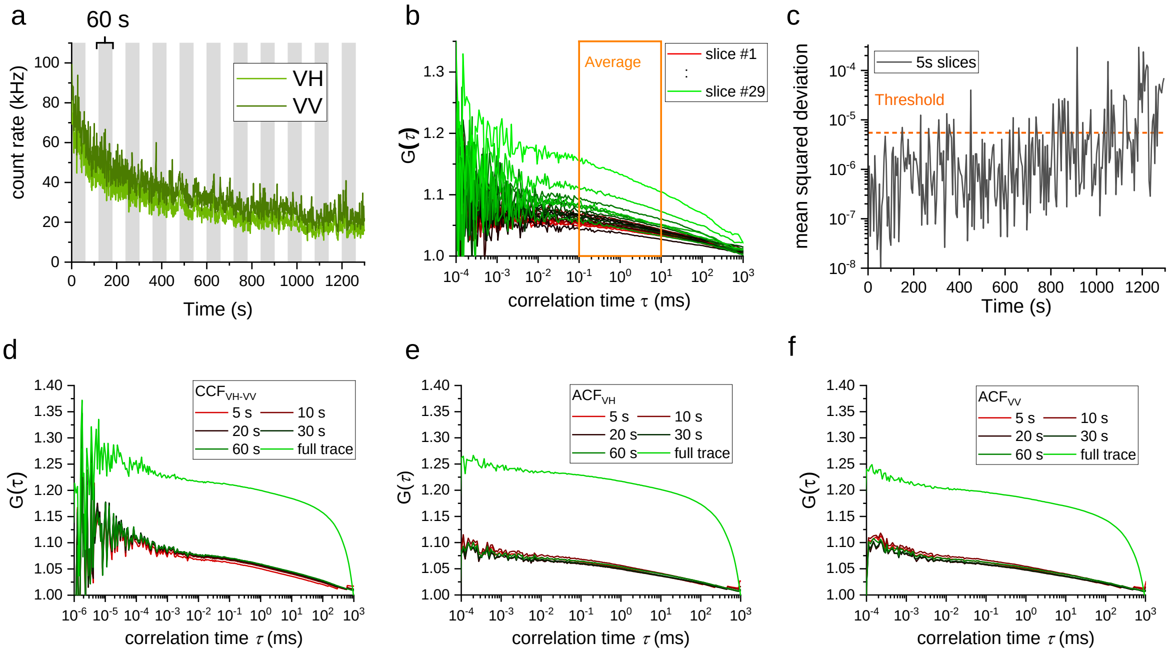

Supplementary Figure 14. Removing photobleaching artefacts by intensity trace splitting. (a) Exemplary photon trace of an NT-EGFP-β_2AR measurement. Each grey and white bar has a width of 60 s and denotes where the photon trace was split into the single slices. (b) Each slice was correlated and the average value A_i in the range from 0.1 – 10 ms was calculated (orange box). (c) The calculated values of A_i were compared to the average of the first n curves (here n = 30) and the mean squared deviation d_i for each curve i was calculated. All curves with a $d_i < d_{max}$ (threshold, orange line, here: 5.5e-6) were averaged and used for further data analysis. (d) Crosscorrelation curves $CCF_{VH\text{-}VV}$ for all time slices and the full trace used as a whole. (e) Autocorrelation curves ACF_{VH} for all time slices and the full trace used as a whole. (f) Autocorrelation curves ACF_{VV} for all time slices and the full trace used as a whole.

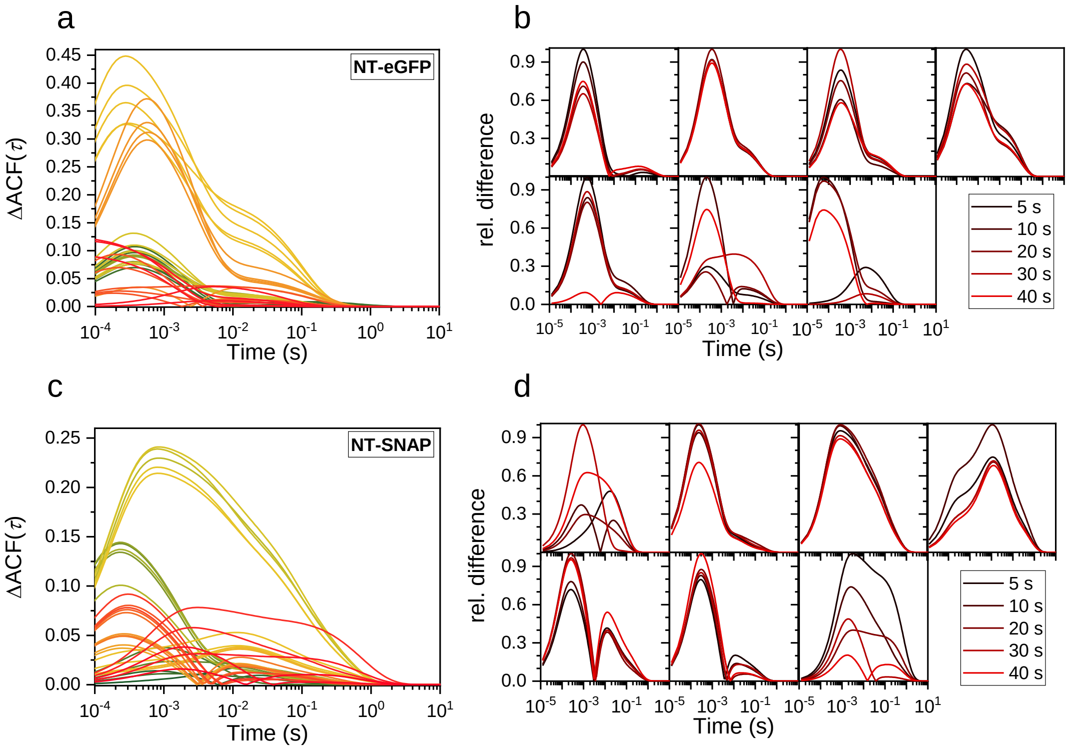

Supplementary Figure 15. Polarization-dependent difference in correlation amplitude found in fullFCS measurements. (a, c) The absolute difference in amplitude between ACF_{VV} and ACF_{VH} calculated from the fit parameter (see Methods) for NT-EGFP (a) and NT-SNAP-β_2-AR (c) measurements. (b, d) For clarity, the absolute difference shown in (a) and (c) are here plotted per sample and were normalized such that the maximal value per sample reached 1.

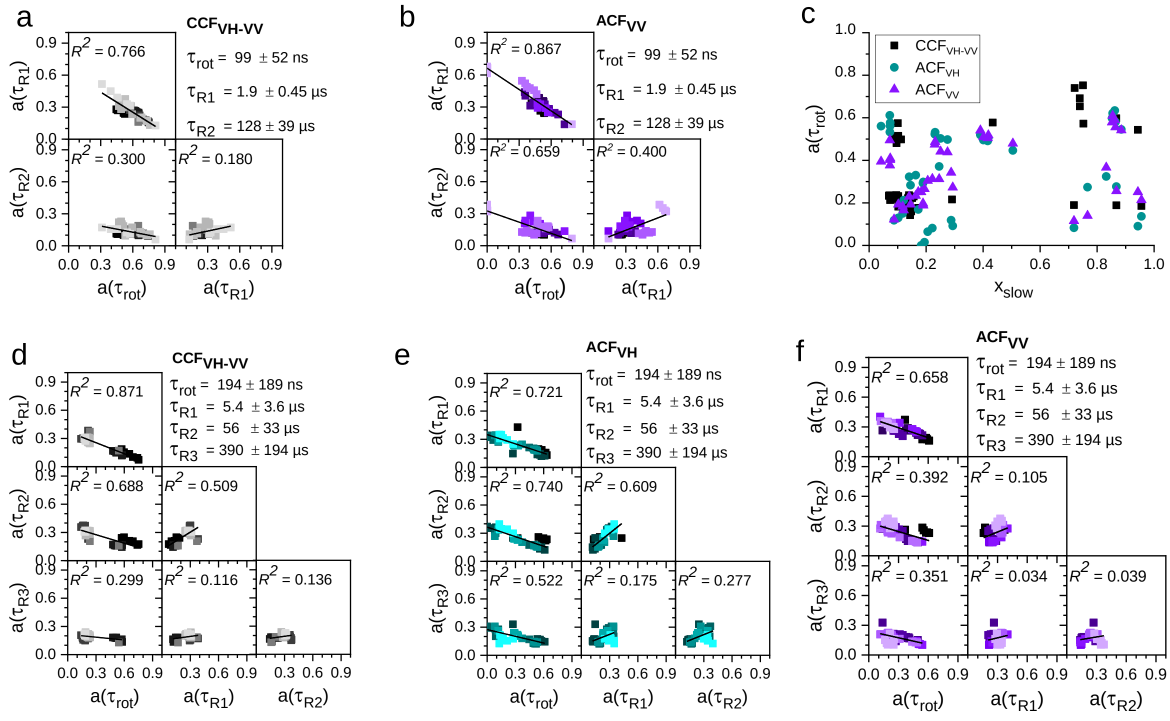

Supplementary Figure 16. NT and S exhibit correlation between different relaxation times. (a, b) Normalized amplitudes of a(τ_{rot}), a(τ_{R1}) and a(τ_{R2}) for the $CCF_{VH\text{-}VV}$ and ACF_{VV} fit results for β_2AR-NT-EGFP. Each color represents the measurement from one cell (n =7). τ_{rot}, τ_{R1} and τ_{R2} are average and standard deviation over all measurements of β_2AR-NT-EGFP. (c) For the β_2AR-NT-SNAP construct no clear dependency of rotational correlation amplitude a(τ_{Rot}) and fraction of slow, membrane diffusion x_{slow} can be seen. (d-f) Normalized amplitudes of a(τ_{rot}), a(τ_{R1}) and a(τ_{R2}) for the $CCF_{VH\text{-}VV}$, ACF_{VH} and ACF_{VV} fit results for β_2AR-NT-SNAP. Each color represents the measurement from one cell (n =7). τ_{rot}, τ_{R1} and τ_{R2} are average and standard deviation over all measurements of β_2AR-NT-SNAP.

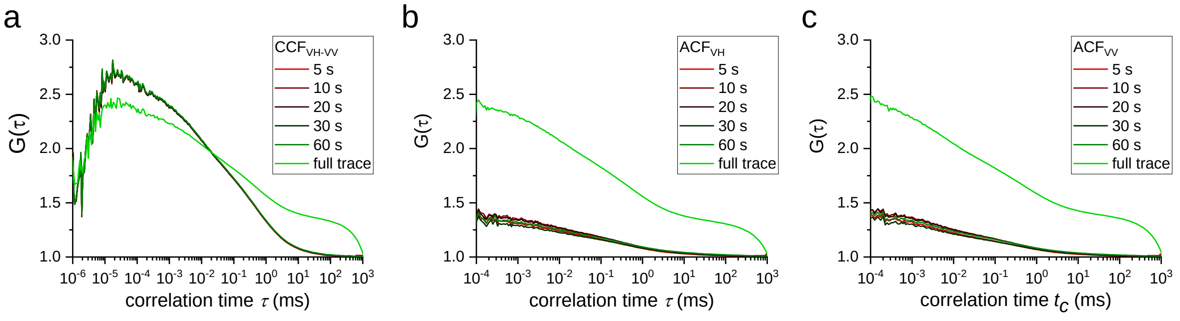

Supplementary Figure 17. fullFCS. Exemplary fullFCS measurement of NT-SNAP-β_2AR construct. (a) Crosscorrelation curves $CCF_{VH\text{-}VV}$ for all time slices and the full trace used as a whole. (b, c) Autocorrelation curves ACF_{VH} and ACF_{VV} for all time slices and the full trace used as a whole.

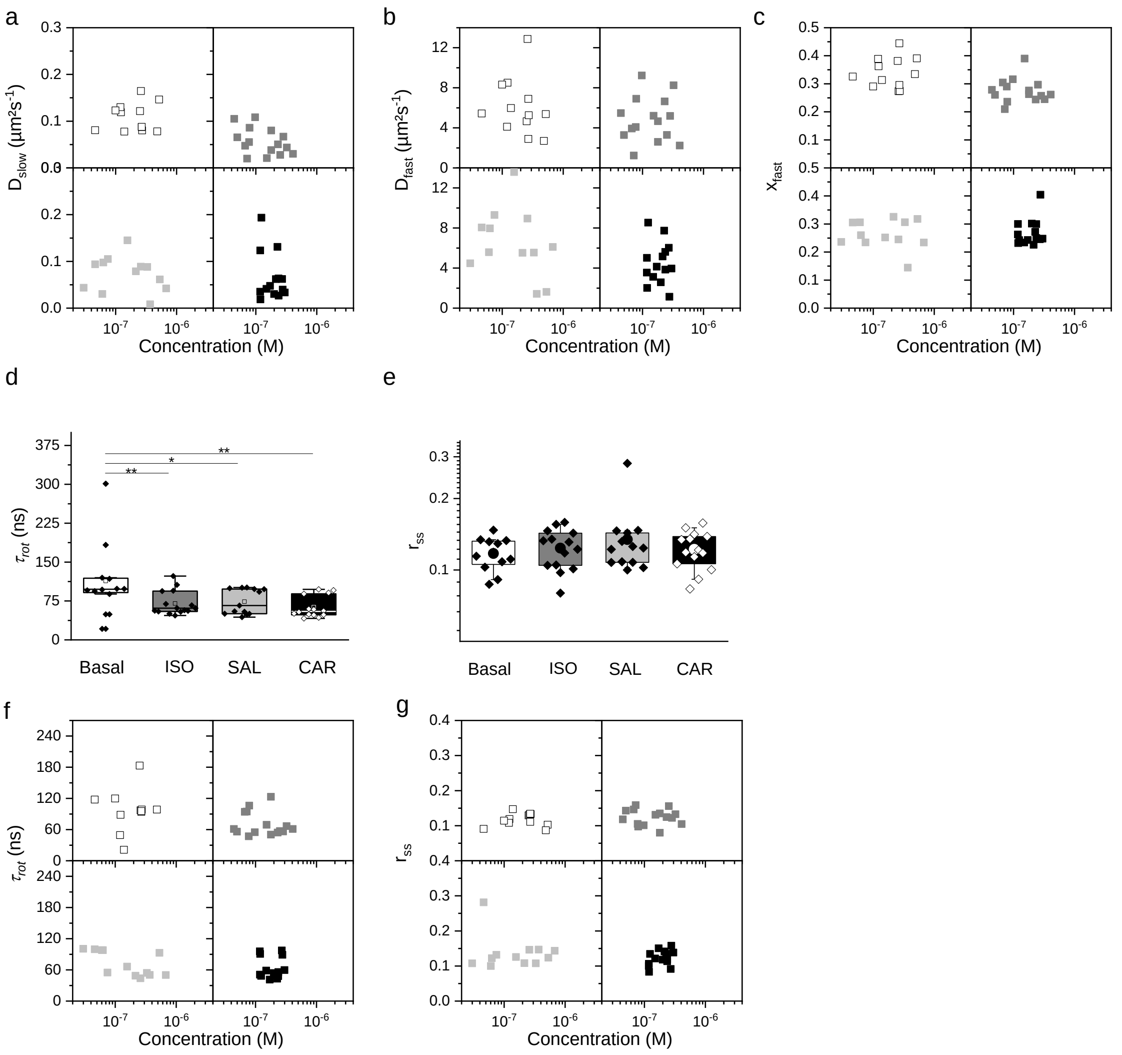

Supplementary Figure 18. Ligand stimulation of NT (a-c, e-g). The mobility measures (slow and fast diffusion coefficient (a, b), fraction of fast diffusion (c) and rotational correlation time (f) and steady-state anisotropy (e,g)) of all ligands tested show largely no dependence on the receptor density in the measured concentration range of 50 nM – 5 µM. White boxes correspond to untreated condition, grey to cells treated with ISO, light grey to cells treated with SAL and black to cells treated with CAR. (d) Rotational correlation times (slow from the biexponential fits) calculated by fitting the photon counting histograms of NT in its basal state and when treated with different ligands. Colour code is the same as in (a). Untreated basal state shows 113 ± 70 ns, Iso shows 70 ± 23 ns, Sal shows 74 ± 24 ns and Car shows 63 ± 21 ns. ** is $P < 0.05$ and * is $P < 0.1$. (e) Distribution of steady state anisotropy r_{ss} of the ligand treated and untreated conditions for NT expressing cells. Colour code is the same as in (a).

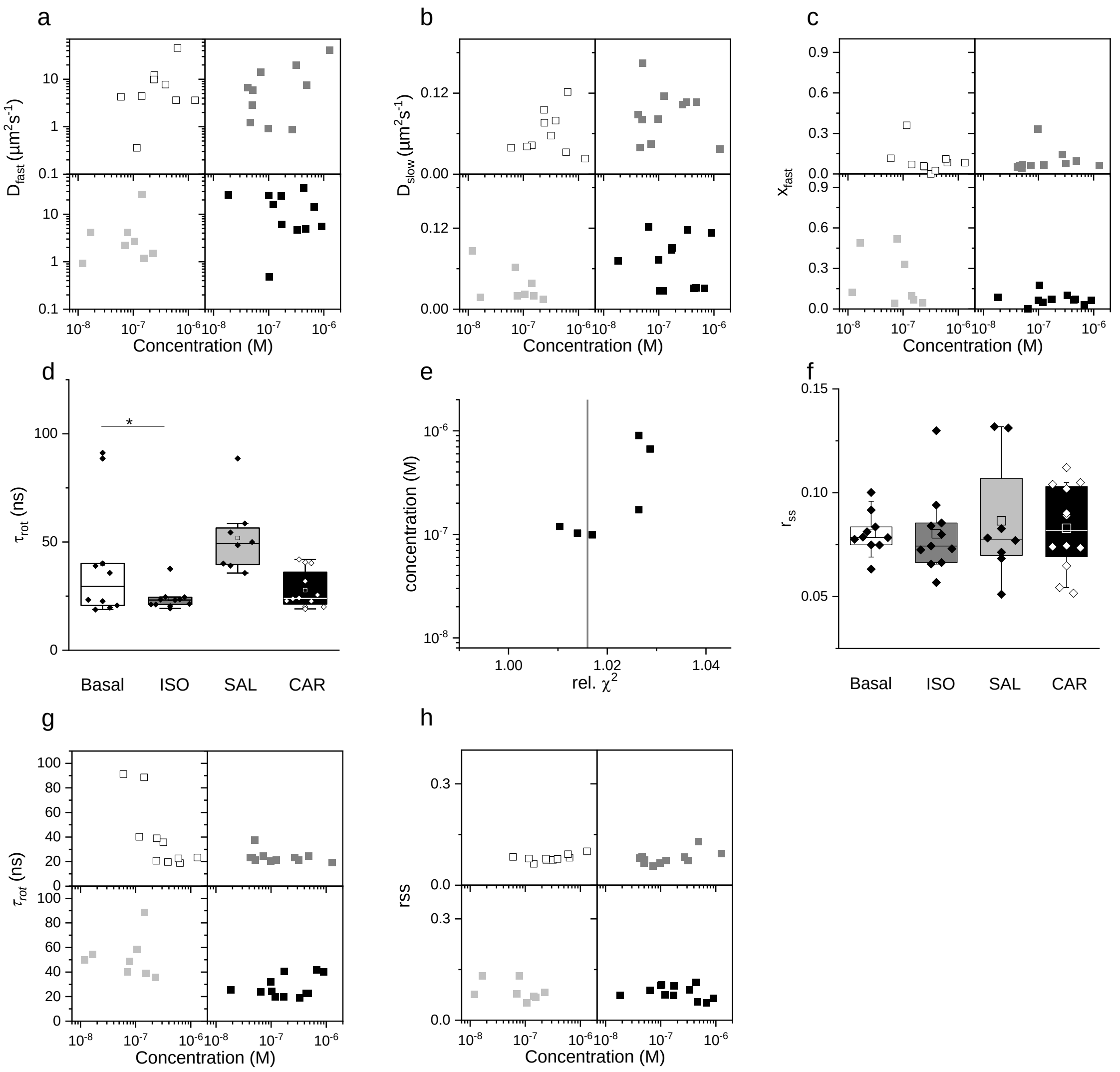

Supplementary Figure 19. Ligand stimulation of S (a-c, f-h). The mobility measures (fast and slow diffusion coefficient (a, b), fraction of fast diffusion (c) and slow rotational correlation time (g) and steady-state anisotropy (f,h) of all ligands tested show largely no clear dependence on the receptor density in the measured concentration range of 10 nM – 5 µM. (d) Rotational correlation times (slowest from the multiexponential fits) calculated by fitting the photon counting histograms of S in its basal state and when treated with different ligands. Colour code is the same as in (a). Untreated basal state shows 40 ± 27 ns, Iso shows 24 ± 5 ns, Sal shows 52 ± 17 ns and Car shows 28 ± 9 ns. * is $P < 0.1$ (e) After CAR stimulation some cells showed strong deviations in the weighted residuals when fitting with a bi-exponential rotational correlation model, thus we analysed all data also with a tri-exponential model, yielding a third fast rotational correlation time. To decide which model to accept as more appropriately describing the data, we (i) inspected the deviations of the weighted residuals and (ii) perform an F-test (1700 data points, 16-18 parameters) with the null-hypothesis "The tri-exponential model describes the data significantly better than the bi-exponential fit". We employed a 2σ-criterion (95 % confidence interval), which brings our threshold line to a relative χ^2 (χ^2_{bi}/χ^2_{tri}) of 1.016. (f) Distribution of steady state anisotropy r_{ss} of the ligand treated and untreated conditions for S expressing cells. Color code is the same as in (a).

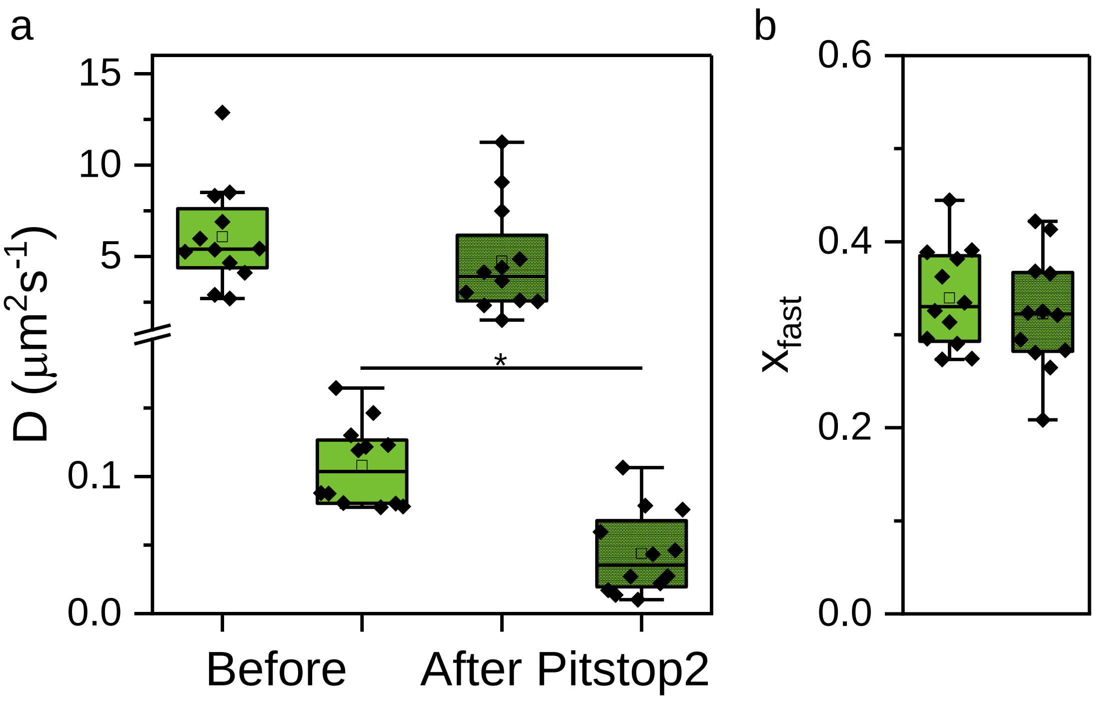

Supplementary Figure 20. Effect of internalization inhibition by Pitstop2. Translational mobility parameters of CHO-K1 cells expressing NT before and after being treated with pitstop2, an inhibitor of receptor internalization. (a) Translational diffusion constants of CHO-K1 cells expressing NT in their basal state and post incubation with pitstop2. Untreated case shows D_{fast} = 6.08 ± 2.80 μm^{2}s^{-1} and D_{slow} = 0.10 ± 0.02 μm^{2}s^{-1}, pitstop2 incubation shows D_{fast} = 4.74 ± 2.99 μm^{2}s^{-1} and D_{slow} = 0.04 ± 0.03 μm^{2}s^{-1}. (b) Fraction of fast diffusion, x_{fast} corresponding to the untreated and pitstop2 treated cells. Untreated cells show 0.33 ± 0.05 and pitstop2 treated cells show 0.32 ± 0.06.

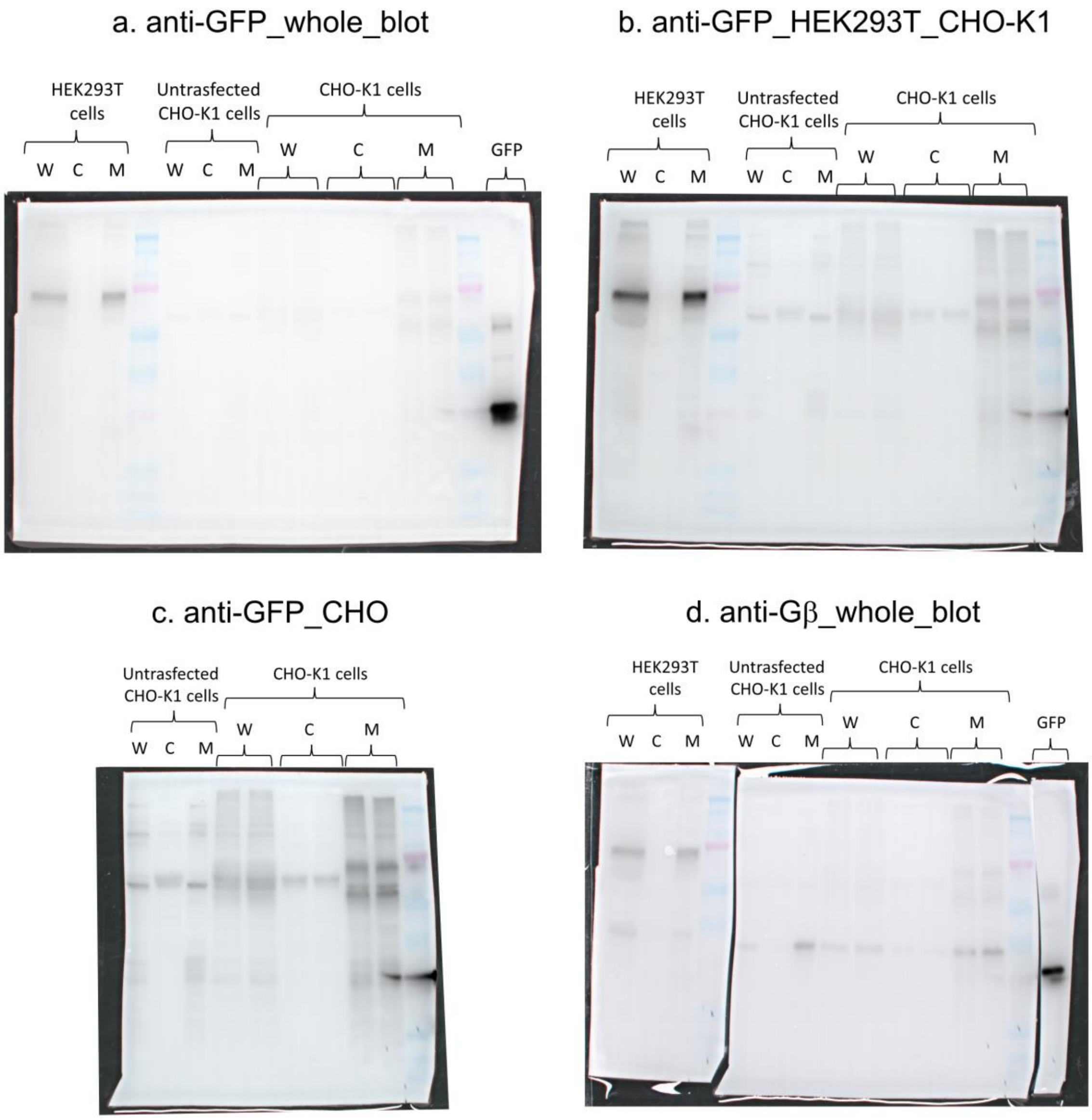

Supplementary Figure 21. Unprocessed western blot of CHO-K1 and HEK293T cells expressing NT stained with anti-GFP and anti-Gβ antibody (The combined blot image is shown in Figure 2d and Supplementary Figure 10). **(a)** Whole blot stained with anti-GFP antibody. **(b)** Blot imaged after the GFP lane in (a) was cut out. **(c)** Blot imaged after the GFP lane and HEK293T sample lanes in (a) were cut out. **(d)** Whole blot stained with anti-Gβ antibody.

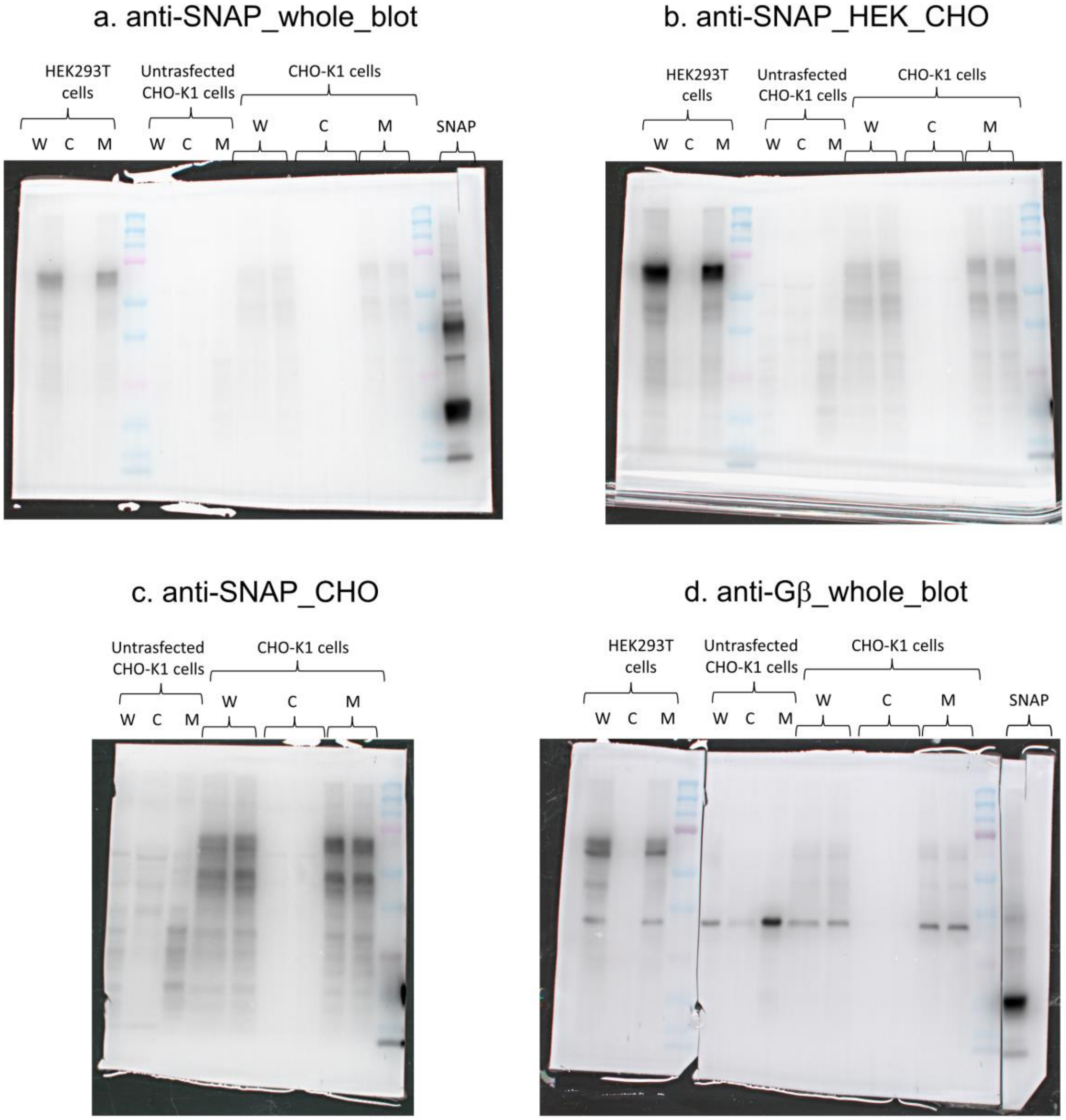

Supplementary Figure 22. Unprocessed western blot of CHO-K1 and HEK293T cells expressing S stained with anti-SNAP and anti-Gβ antibody (The combined blot image is shown in Supplementary Figure 11). **(a)** Whole blot stained with anti-SNAP antibody. **(b)** Blot imaged after the SNAP lanes in (a) was cut out. **(c)** Blot imaged after the SNAP lanes and HEK293T sample lanes in (a) were cut out. **(d)** Whole blot stained with anti-Gβ antibody.

Supplementary Table 1. EC_{50} values obtained from Fluorimetric cAMP assay shown in Figure S1.

Construct	EC_{50} (nM)
WT	1.3 ± 0.8
NT	4.9 ± 3.7
IL3	32.9 ± 19.5
WT (HEK293T)	15.3 ± 3.8
TAG	2.1 ± 0.0
WT_A	88.0 ± 66.3
NT_A	39.1 ± 37.5

Note: Data are mean ± SEM from the fit using eq. 1. The data points for the fit were acquired from three independent experiments. NT and IL3 are not statistically significant from WT for $P < 0.05$. TAG is statistically significant from WT for $P < 0.1$, but it has to be noted that the fit for TAG gave a R^2 of 0.94 relative to 0.99 of WT (HEK293T). Also both WT (HEK293T) and TAG are saturated at 100 nM ligand concentration. NT_A is not statistically significant from WT_A for $P < 0.05$.

Supplementary Table 2. Summary fit results FCS. Summary of the fit results for all constructs measured within this study. For FCS, data from the 2D3DT model (eq. 4) is shown.

Construct	N_{cells}	N	τ_{D1} (ms)	x_{fast}	D_{fast} ($\mu m^2 s^{-1}$)	τ_{D2} (ms)	D_{slow} ($\mu m^2 s^{-1}$)
**NT**	12	59.9 ± 36.9	2.71± 1.21	0.33 ± 0.05	6.08 ± 2.80	136.6 ± 35.2	0.10 ± 0.02
**NT (HEK293T)**	10	58.02 ± 6.76	2.37 ± 1.61	0.36 ± 0.07	8.35 ± 5.34	93.1 ± 63.4	0.25 ± 0.18
**IL3**	12	89.1 ± 115.5	2.01 ± 0.87	0.31 ± 0.04	8.09 ± 3.65	128.5 ± 59.0	0.13 ± 0.06
**S**	10	75.2 ± 58.7	1.88 ± 1.03	0.07 ± 0.03	11.0 ± 13.2	25.0 ± 23.9	0.06 ± 0.03
**TAG**	6	44.7 ± 31.1	3.08 ± 0.75	0.27 ± 0.10	4.74 ± 1.36	243.7 ± 53.5	0.06 ± 0.01
**NT-A**	19	77.7 ± 59.4	1.83 ± 0.89	0.34 ± 0.07	6.29 ± 3.18	172.0 ± 111.8	0.08 ± 0.08
**S-A**	22	164.5 ± 101.8	0.69 ± 0.89	0.10 ± 0.05	43.3 ± 34.3	101.7 ± 55.7	0.11 ± 0.05

Supplementary Table 3. Summary fit results TRA. Summary of the fit results for all constructs measured within this study. For TAG and S also the number of constructs, which required a bi-exponential fit is shown. The average rotational correlation time τ_{rot} was calculated based on the longer τ_{rot} neglecting the fast component of free dye rotation.

Construct	N_{cells}	τ_{rot} (ns)	$\tau_{rot,fast1}$ (ns)	$\tau_{rot,fast2}$ (ns)	N_{fast1}	N_{fast2}	x_{fast1}	x_{fast2}	r_{ss}
**NT**	12	113.7 ± 70.5	0.04 ± 0.02	-	12	0	0.81 ± 0.11-	-	0.10 ± 0.02
**NT (HEK293T)**	10	58.4 ± 54	0.58 ± 1.15	2.2 ± 1.2	0	10	0.34 ± 0.18	0.18 ± 0.04	0.13 ± 0.02
**IL3**	12	165.1 ± 14.6	0.06 ± 0.02-	-	12	0	0.69 ± 0.10-	-	0.13 ± 0.02
**S**	10	40.0 ± 27.5	0.06 ± 0.03	-	10	0	0.56 ± 0.07	-	0.08 ± 0.01
**TAG**	6	118.3 ± 51.8	0.82 ± 0.55	0.09 ± 0.03	4	2	0.32 ± 0.02	0.41 ± 0.10	0.10 ± 0.01
**NT-A**	19	177.0 ± 20.8	-	-	0	0	-	-	0.10 ± 0.01
**S-A**	22	50.3 ± 9.2	0.27 ± 0.20	-	4	-	0.20 ± 0.19	-	0.10 ± 0.01

Supplementary Table 4. Summary fit results fullFCS. Summary of the fit results for continuous-wave FCS measurements collected within this study. Crosscorrelation data was fitted with the additional photon-antibunching term (eq. 10), while autocorrelations were fitted with the 2D3DT model, where two or three additional relaxation times have been added (eq. 4). Note that the data was fitted globally per sample, only fractions were left to vary (see Methods).

**Construct**	**Correlation**	**N**	τ_{rot} **(ns)**	x_{rot}	τ_{R1} **(μs)**	x_{R1}	τ_{R2} **(μs)**	x_{R2}	τ_{R3} **(μs)**	x_{R3}	τ_{D1} **(ms)**	x_{D1}	τ_{D2} **(ms)**
**NT**	**CCF_{VH-VV}**	10.7± 6.9	99 ± 52	0.52 ± 0.18	1.9 ± 0.45	0.26 ± 0.08	128 ± 39	0.13 ± 0.04	-	-	2.04 ± 1.34	0.35 ± 0.08	109.3 ± 112.4
	**ACF_{VH}**			0.39 ± 0.27		0.39 ± 0.19		0.20 ± 0.11		-			
	**ACF_{VV}**			0.44 ± 0.01		0.37 ± 0.12		0.17 ± 0.06		-			
**S**	**CCF_{VH-VV}**	3.54 ± 3.91	194 ± 189	0.33 ± 0.19	5.4 ± 3.6	0.24 ± 0.08	56 ± 33	0.27 ± 0.07	390 ± 194	0.19 ± 0.02	1.64 ± 0.98	0.71 ± 0.30	119.0 ± 247.1
	**ACF_{VH}**			0.35 ± 0.20		0.24 ± 0.08		0.25 ± 0.08		0.20 ± 0.06			
	**ACF_{VV}**			0.35 ± 0.06		0.27 ± 0.11		0.23 ± 0.06		0.17 ± 0.06			

Supplementary Table 5. Summary fit results of FCS after ligand stimulation. Summary of the fit results for all ligand-stimulated measurements within this study. For FCS, data from the 2D3DT model (eq. 4) is shown.

Ligand	Construct	N_{cells}	N	τ_{D1} (ms)	x_{fast}	D_{fast} (μm^{2}s^{-1})	τ_{D2} (ms)	D_{slow} (μm^{2}s^{-1})	D_{AVG} (μm^{2}s^{-1})
**Iso**	NT	15	25.0 ± 16.9	2.98 ± 1.94	0.27 ± 0.04	4.81 ± 2.23	256.3 ± 144.8	0.06 ± 0.03	0.04 ± 0.02
	S	11	37.8 ± 53.3	11.6 ± 85.4	0.10 ± 0.08	18.6 ± 30.6	240.8 ± 211.5	0.09 ± 0.04	0.08 ± 0.03
**Sal**	NT	13	32.6 ± 31.5	2.53 ± 2.24	0.26 ± 0.04	6.68 ± 3.28	260.5 ± 346.4	0.07 ± 0.03	0.06 ± 0.03
	S	8	18.4 ± 15.2	4.22 ± 3.44	0.30 ± 0.25	5.95 ± 7.74	354.8 ± 212.5	0.04 ± 0.03	0.03 ± 0.02
**Car**	NT	14	31.1 ± 9.19	3.32 ± 2.23	0.26 ± 0.04	4.45 ± 2.07	260.9 ± 149.6	0.06 ± 0.05	0.05 ± 0.04
	S	12	51.0 ± 47.3	3.32 ± 7.25	0.08 ± 0.04	14.7 ± 11.5	227.4 ± 140.0	0.07 ± 0.04	0.06 ± 0.03

Supplementary Table 6. Summary fit results of TRA after ligand stimulation. Summary of the fit results for all constructs measured within this study. Some samples required a bi-exponential fit. The average rotational correlation time τ_{rot} was calculated based on the longer τ_{rot} neglecting the fast component of free dye rotation.

Ligand	Construct	N_{cells}	τ_{rot} (ns)	$\tau_{rot,fast1}$ (ns)	$\tau_{rot,fast2}$ (ns)	N_{fast1}	N_{fast2}	x_{fast1}	x_{fast2}	r_{ss}
**Iso**	**NT**	15	70.1 ± 23.0	0.16 ± 0.25	-	15	0	0.24 ± 0.11	-	0.12 ± 0.02
	**S**	11	23.6 ± 4.9	0.09 ± 0.04	-	11	0	0.55 ± 0.12	-	0.08 ± 0.02
**Sal**	**NT**	13	73.7 ± 24.3	0.26 ± 0.11	-	13	0	0.27 ± 0.11	-	0.13 ± 0.04
	**S**	8	51.8 ± 16.8	0.08 ± 0.06	-	8	0	0.56 ± 0.09	-	0.09 ± 0.03
**Car**	**NT**	14	63.0 ± 20.6	0.30 ± 0.89	-	14	0	0.25 ± 0.12	-	0.12 ± 0.02
	**S**	12	27.7 ± 8.62	0.82 ± 1.20	0.02 ± 0.02	10	2	0.38 ± 0.24	0.48 ± 0.12	0.08 ± 0.02

Supplementary References

1 Wieland, K., Zuurmond, H. M., Krasel, C., Ijzerman, A. P. & Lohse, M. J. Involvement of Asn-293 in stereospecific agonist recognition and in activation of the beta 2-adrenergic receptor. *Proceedings of the National Academy of Sciences* **93**, 9276, doi:10.1073/pnas.93.17.9276 (1996).
