# Supplementary material for: Unraveling the hidden temporal range of fast β2-adrenergic receptor mobility by time-resolved fluorescence
Source: Commun Biol. 2022 Feb 28;5:176. doi: 10.1038/s42003-022-03106-4 (PMC8885909; doi:10.1038/s42003-022-03106-4)
Supplement: Supplementary file 3 — Description of Additional Supplementary Files [file 42003_2022_3106_MOESM3_ESM.pdf]

Description of Additional Supplementary Files

File name: Supplementary Data 1

Description: Source data underlying the graphs in the main figures (.zip folder).

- **Figure 2 files:**

2a_ACF_rep.txt	FCS curve, fit and residual for the representative ACF in Figure 2a
2b_D_b2ar.txt	Diffusion constants of all β_2-AR constructs used in the study
2c_x_fast_b2ar.txt	Fraction of the fast diffusion constant from all β_2-AR constructs used in the study
2d_Focus_change.txt	Fraction of the fast diffusion constant acquired from different depths in a cell

- **Figure 3 files:**

3a_TRA_fit.txt	Fluorescence decays of both parallel and perpendicular detection channel with the associated IRFs. The fits for the decay and the corresponding residuals are given. Anisotropy decay and fit is also given.
3b_t_rot_b2ar.txt	Slow rotational correlation times from TRA fits of all β_2-AR constructs used in the study
3c_r_ss_conc_b2ar.txt	Steady state anisotropy as a function of receptor concentration corresponding to all β_2-AR constructs used in the study

- **Figure 4 files:**

4a_fullfcs_fit.txt	ACFs and CCF of a representative fullFCS measurement with its corresponding fits and residuals
4b_dACF.txt	The absolute difference in ACFs to signify the presence of rotational dynamics
4c_scatter, 4c_scatter_fits.txt	Relation between the different relaxation times and rotational correlation times
4d_x_slow_v_t_rot_scatter.txt, 4d_x_slow_v_t_rot_fit.txt	The fraction of the slow diffusion constant expressed as a function of the corresponding rotational correlation time

- **Figure 5 files:**

5a_NT_D_avg.txt	Weighted slow diffusion constant of NT in its untreated basal state and ligand treated state
5b_S_D_avg.txt	Weighted slow diffusion constant of S in its untreated basal state and ligand treated state
