# Supplementary material for: Unraveling the hidden temporal range of fast β2-adrenergic receptor mobility by time-resolved fluorescence
Source: Commun Biol. 2022 Feb 28;5:176. doi: 10.1038/s42003-022-03106-4 (PMC8885909; doi:10.1038/s42003-022-03106-4)
Supplement: Supplementary file 4 — Supplementary Data 1 [file 42003_2022_3106_MOESM4_ESM.zip › SupplementaryData/Readme_supplementary_data.docx]

**Supplementary data of graphs in main figures**

| 2a_ACF_rep.txt | FCS curve, fit and residual for the representative ACF in Figure 2a |
| --- | --- |
| 2b_D_b2ar.txt | Diffusion constants of all β_2_-AR constructs used in the study |
| 2c_x_fast_b2ar.txt | Fraction of the fast diffusion constant from all β_2_-AR constructs used in the study |
| 2d_Focus_change.txt | Fraction of the fast diffusion constant acquired from different depths in a cell |
| 3a_TRA_fit.txt | Fluorescence decays of both parallel and perpendicular detection channel with the associated IRFs. The fits for the decay and the corresponding residuals are given. Anisotropy decay and fit is also given. |
| 3b_t_rot_b2ar.txt | Slow rotational correlation times from TRA fits of all β_2_-AR constructs used in the study |
| 3c_r_ss_conc_b2ar.txt | Steady state anisotropy as a function of receptor concentration corresponding to all β_2_-AR constructs used in the study |
| 4a_fullfcs_fit.txt | ACFs and CCF of a representative fullFCS measurement with its corresponding fits and residuals |
| 4b_dACF.txt | The absolute difference in ACFs to signify the presence of rotational dynamics |
| 4c_scatter, 4c_scatter_fits.txt | Relation between the different relaxation times and rotational correlation times |
| 4d_x_slow_v_t_rot_scatter.txt , 4d_x_slow_v_t_rot_fit.txt | The fraction of the slow diffusion constant expressed as a function of the corresponding rotational correlation time |
| 5a_NT_D_avg.txt | Weighted slow diffusion constant of NT in its untreated basal state and ligand treated state |
| 5b_S_D_avg.txt | Weighted slow diffusion constant of S in its untreated basal state and ligand treated state |
