# Supplementary material for: Unraveling the hidden temporal range of fast β2-adrenergic receptor mobility by time-resolved fluorescence
Source: Commun Biol. 2022 Feb 28;5:176. doi: 10.1038/s42003-022-03106-4 (PMC8885909; doi:10.1038/s42003-022-03106-4)
Supplement: Supplementary file 5 — Reporting Summary [file 42003_2022_3106_MOESM5_ESM.pdf]

nature portfolio

Corresponding author(s): Prof. Dr. Katrin G. Heinze
Prof. Dr. Martin J. Lohse

Last updated by author(s): Dec 16, 2021

Reporting Summary

Nature Portfolio wishes to improve the reproducibility of the work that we publish. This form provides structure for consistency and transparency in reporting. For further information on Nature Portfolio policies, see our Editorial Policies and the Editorial Policy Checklist.

Statistics

For all statistical analyses, confirm that the following items are present in the figure legend, table legend, main text, or Methods section.

n/a	Confirmed	
☐	☒	The exact sample size (*n*) for each experimental group/condition, given as a discrete number and unit of measurement
☐	☒	A statement on whether measurements were taken from distinct samples or whether the same sample was measured repeatedly
☐	☒	The statistical test(s) used AND whether they are one- or two-sided<br>*Only common tests should be described solely by name; describe more complex techniques in the Methods section.*
☒	☐	A description of all covariates tested
☒	☐	A description of any assumptions or corrections, such as tests of normality and adjustment for multiple comparisons
☐	☒	A full description of the statistical parameters including central tendency (e.g. means) or other basic estimates (e.g. regression coefficient) AND variation (e.g. standard deviation) or associated estimates of uncertainty (e.g. confidence intervals)
☐	☒	For null hypothesis testing, the test statistic (e.g. *F*, *t*, *r*) with confidence intervals, effect sizes, degrees of freedom and *P* value noted<br>*Give P values as exact values whenever suitable.*
☒	☐	For Bayesian analysis, information on the choice of priors and Markov chain Monte Carlo settings
☒	☐	For hierarchical and complex designs, identification of the appropriate level for tests and full reporting of outcomes
☒	☐	Estimates of effect sizes (e.g. Cohen's *d*, Pearson's *r*), indicating how they were calculated

Our web collection on statistics for biologists contains articles on many of the points above.

Software and code

Policy information about availability of computer code

Data collection	SymPhoTime64 version 2.7 was used for the time-resolved measurements, Leica LAS X was used for imaging.
Data analysis	SymPhoTime64 version 2.7 was used for correlation of the time-resolved fluorescence spectroscopic data and exporting fluorescence decay histograms. Analysis of the correlation curves were performed on Origin Pro 2020. Global fit of the fluorescence decay histograms was performed on MATLAB R2020b using custom scripts. Custom python scripts were used for split correlation of the fullFCS data, https://github.com/khemmen/katcorr/; DOI: 10.5281/zenodo.5786498. ChiSurf version 19.07.08 was used for analyis of the fullFCS data.

For manuscripts utilizing custom algorithms or software that are central to the research but not yet described in published literature, software must be made available to editors and reviewers. We strongly encourage code deposition in a community repository (e.g. GitHub). See the Nature Portfolio guidelines for submitting code & software for further information.

Data

Policy information about availability of data

All manuscripts must include a data availability statement. This statement should provide the following information, where applicable:

- Accession codes, unique identifiers, or web links for publicly available datasets
- A description of any restrictions on data availability
- For clinical datasets or third party data, please ensure that the statement adheres to our policy

All data associated with the manuscript has been used in the manuscript. The source data for the graphs in the main figures are available as supplementary data and the raw acquisition data is available upon reasonable request from the corresponding authors. The custom scripts used for split correlation is available on github at https://
github.com/khemmen/katcorr/; DOI: 10.5281/zenodo.5786498.

Field-specific reporting

Please select the one below that is the best fit for your research. If you are not sure, read the appropriate sections before making your selection.

☒ Life sciences ☐ Behavioural & social sciences ☐ Ecological, evolutionary & environmental sciences

For a reference copy of the document with all sections, see nature.com/documents/nr-reporting-summary-flat.pdf

Life sciences study design

All studies must disclose on these points even when the disclosure is negative.

Sample size	No statistical methods were used to determine sample size.
Data exclusions	No data were excluded from the manuscript.
Replication	Data was acquired over different days to replicate a measurement.
Randomization	The experiments were not randomized. Randomization is not relevant as each measurement was grouped based on the construct. Cell measurements from a given construct were clubbed together.
Blinding	Blinding is not relevant here, as time-resolved fluorescence spectroscopy does not require blind allocation of groups.

Reporting for specific materials, systems and methods

We require information from authors about some types of materials, experimental systems and methods used in many studies. Here, indicate whether each material, system or method listed is relevant to your study. If you are not sure if a list item applies to your research, read the appropriate section before selecting a response.

Materials & experimental systems

n/a	Involved in the study	
☐	☒	Antibodies
☐	☒	Eukaryotic cell lines
☒	☐	Palaeontology and archaeology
☒	☐	Animals and other organisms
☒	☐	Human research participants
☒	☐	Clinical data
☒	☐	Dual use research of concern

Methods

n/a	Involved in the study	
☒	☐	ChIP-seq
☒	☐	Flow cytometry
☒	☐	MRI-based neuroimaging

Antibodies

Antibodies used	anti-GFP (Abcam order no. ab32146, lot:GR253725-19); anti-β2AR (Abcam order no. ab61778, GR3249046-5); anti-SNAP-tag (NEB, order no. P9310S); anti-Gβ (Santa Cruz Biotechnology order no. sc-166123); HRP Goat Anti-Rabbit (Abcam order no. ab205718)
Validation	The validation has been given in the manufacturer's website, https://www.abcam.com/gfp-antibody-e385-ab32146.html for the anti-GFP antibody; https://www.abcam.com/beta-2-adrenergic-receptor-antibody-ab61778.html for the anti-β2AR antibody; https://international.neb.com/products/p9310-anti-snap-tag-antibody-polyclonal#Product%20Information for the anti-SNAP-tag antibody and https://www.scbt.com/p/gbeta-antibody-h-1 for the anti-Gβ antibody.

Eukaryotic cell lines

Policy information about cell lines

Cell line source(s)	CHO-K1 and HEK293T were from ATCC as mentioned in "Materials and Methods"
Authentication	Authenticated by the supplier, https://www.atcc.org/products/ccl-61, https://www.atcc.org/products/crl-3216
Mycoplasma contamination	CHO-K1 tested negative to mycoplasma. HEK293T was not tested for mycoplasma, fetal calf serum used in the media for all cells tested negative for mycoplasma.
Commonly misidentified lines (See ICLAC register)	The used cell lines are not listed in the database of commonly misidentified cell lines.
